# Modification of the asthma quality of life questionnaire (standardised) for patients 12 years and older

**DOI:** 10.1186/1477-7525-3-58

**Published:** 2005-09-16

**Authors:** Elizabeth F Juniper, Klas Svensson, Ann-Christin Mörk, Elisabeth Ståhl

**Affiliations:** 1 Department of Clinical Epidemiology and Biostatistics, McMaster University, Hamilton, Canada; 2 AstraZeneca R&D, Lund, Sweden

## Abstract

**Background:**

The age limit for some adult asthma clinical trials has recently been lowered to 12 years. In this study we have made minor modifications to the standardised version of the adult Asthma Quality of Life Questionnaire (AQLQ(S)) to make it valid for patients 12 years and older (AQLQ12+).

**Methods:**

We have used two clinical trial databases, in which the AQLQ12+ was used, to compare the measurement properties of the questionnaire in patients 12–17 years and patients 18 years and older. A total of 2433 patients (12–75 years), with current asthma and with data that could be evaluated both at randomisation and end of treatment, were included.

**Results:**

The analysis showed that internal consistency, responsiveness and correlations with other clinical indices were very similar in patients 12–17 years and patients 18 years and older.

**Conclusion:**

The measurement properties of the AQLQ12+ are similar in adolescents and adults and therefore the instrument is valid for use in adult studies which include children 12 years and older.

## Background

The Asthma Quality of Life Questionnaire (AQLQ) was developed to measure the functional impairments experienced by adults 17 years and older [1]. It has 32 items in four domains (symptoms, activity limitations, emotional function and environmental stimuli). In the original AQLQ, five of the activity questions are patient-specific but this proved time-consuming and troublesome in large clinical trials. To address this problem, we developed the standardised version (AQLQ(S)). In the AQLQ(S) all the activity questions are generic and its measurement properties that are almost the same as those of the original AQLQ [3]. The Paediatric Asthma Quality of Life Questionnaire was developed to measure the problems that children 7–17 years experience [2]. It has 23 items in three domains (symptoms, activity limitations and emotional function). Recently, adolescents 12 years and older have been included in adult asthma clinical trials. To avoid using two separate health-related quality of life questionnaires in these studies, we have modified the AQLQ(S) [3] to make it suitable for both adolescents and adults. The AQLQ(S) was selected in preference to the original AQLQ because it is the version of the questionnaire most commonly used in clinical trials.

The aim of this adaptation was to ensure that the problems that are most troublesome to adolescents were included whilst making as few modifications to the original as possible. We have used two clinical trial databases to compare the measurement properties of the AQLQ(S) for 12 years and older (AQLQ12+) in patients 12–17 years and patients ≥ 18 years.

## Methods

### Modification of the AQLQ(S)

Both the AQLQ [1] and the Paediatric Asthma Quality of Life Questionnaire (PAQLQ) [2] were developed by asking adults and children respectively about the problems and limitations that are most important to them in their daily lives as a result of their asthma. Items that were most frequently experienced and most troublesome for the two groups and which are included in the two questionnaires [1,2] are shown in Tables 1 and 2. After reviewing the two questionnaires, only one word needed to be added to the AQLQ(S) to make it suitable for adolescents 12 years and older. As can be seen in Tables 1 and 2, symptoms and activity limitations are very similar in adults and children and the only change necessary was to alter the activity question about 'work-related limitations' to ask about 'work-/school-related limitations'. Although children identified sleep as a troublesome activity, this is already included in the symptom domain of the AQLQ. There is no environmental stimuli domain in the PAQLQ because children tend to express their problems with the environment in terms of activity limitation. For instance, an adult will say 'I am bothered by cigarette smoke', a young child will say 'I can't go to my friend's house because her mum smokes'. We considered that as adolescents (12–17 years) are moving towards adulthood, they would be old enough to express directly, rather than indirectly, their problems with environmental stimuli. Although children experience similar fears and frustrations to adults, they also 'feel different and left out'. Since none of the emotional function questions in the AQLQ could be modified to take this into account and since adding a separate question would have altered the weighting of the domain and overall score, 'feel different and left out' has not been included in the AQLQ12+.

**Table 1:** Functional impairments most important to adults (17–70 years) (1)

**Symptoms**	**Emotions**	**Activities**	**Environment**
Short of breath	Afraid of not having medications available	Exercise/sports	Cigarettes
Chest tightness		Hurrying	Dust
Wheeze	Afraid of getting out of breath	Social activities	Air pollution
Cough	Concerned about the need to use medications	Pets	Cold air
Tired	Frustrated	Work/housework	Pollen

**Table 2:** Functional impairments most important to children (7–17 years) (2)

**Symptoms**	**Activities**	**Emotions**
Short of breath	Sports and games	Feel different and left out
Chest tightness	Activities with friends	Frustrated
Cough	Playing with pets	Angry
Wheeze	School activities	Sad
Tired	Sleeping	Frightened/anxious

### Studies and patients

The analysis was conducted using databases from two clinical trials. Full details of one trial have been published elsewhere [4]. The second trial has been published as abstracts [5,6]. The first trial was a 12-month, randomised, double-blind, parallel group study comparing two active interventions. Of the 1890 patients randomised, 1770 completed the AQLQ12+ at baseline and either at the end of 12 months or on withdrawal. The second study was a 12-week randomised trial comparing three active interventions. Of the 680 patients randomised, 655 completed the AQLQ12+ at randomisation and either at 12 weeks or withdrawal. In both studies, patients were required to have inadequately controlled asthma with no evidence of any other respiratory disease and to be between the ages of 12 and 80 years.

### Outcomes

#### Asthma Quality of Life Questionnaire for 12 years and older (AQLQ12+)

Patients are asked to recall their experiences during the previous 2 weeks and to score each of the 32 questions on a 7-point scale from 7 = no impairment to 1 = severe impairment. The overall score is calculated as the mean response to all questions. The four domain scores (symptoms, activity limitations, emotional function and environmental stimuli) are the means of the responses to the questions in each of the domains.

#### Symptom and Medication Diary

Each morning and evening patients scored how much they were bothered by their asthma symptoms on a 4-point scale (0 = no symptoms and 3 = unable to do normal daily activities (or sleep) because of asthma) and recorded the amount of rescue medication taken. Each morning they recorded whether they had been woken during the night by asthma symptoms. They also measured pre-bronchodilator PEF each morning and evening, recording the best of three blows. For this analysis, we have estimated the mean diary scores for the 2 weeks that were co-incident with the AQLQ12+ two week recall period.

#### Spirometry

Pre-bronchodilator FEV_1_% predicted normal was recorded at all clinic visits.

### Statistical analysis

Since the validity of the AQLQ(S) has already been established in adults and because only one word was added to the AQLQ(S) for the modification, AQLQ12+ scores for adults (18–80 years) have been considered the gold standard for this analysis (criterion validity). Data collected at baseline were used to determine differences between age groups (unpaired t-test) and internal consistency (Cronbach's alpha). Change in scores between baseline and end of treatment, adjusted for treatment effect and baseline values by a linear ANOVA model, were used to determine responsiveness. Cross-sectional and longitudinal construct validity were evaluated by examining Pearson correlation coefficients between the AQLQ12+ and both diary symptoms and airway calibre.

## Results

2423 patients were included in the analysis. There were 2207 over 18 years and 216 between 12 and 17 years (Table 3). In the older patients there were slightly more women than men and in the younger patients slightly more men than women. FEV_1_% predicted was slightly higher in the younger patients.

**Table 3:** Demographics and baseline values

	**Study 1**		**Study 2**	
	≥ 18 years	12–17 years	≥ 18 years	12–17 years
Number of patients	1652	116	555	100
Mean age (range)	44.8 (18–80)	14.3 (12–17)	44.6 (18–79)	13.9 (12–17)
Gender M/F (%)	41.1/58.9	57.8/42.2	33.5/66.5	53.0/47.0
FEV_1_% pred. (range)	75.4 (32–136)	83.9 (47–125)	73.3 (41–107)	77.8 (54–114)

At baseline in both studies, there was no evidence of any difference in AQLQ12+ scores both for overall AQLQ12+ scores and for the symptom and emotional function domain scores (Table 4). However, in study 1, 12–17 year old patients were less troubled by activity limitations and environmental stimuli than older patients but the differences were small (< 0.35) and cannot be considered of clinical importance [5]. These differences were not seen in study 2. After adjusting for treatment and baseline values, changes in AQLQ12+ scores during the treatment period were similar in the two age groups for both studies (Table 4). As further evidence of the validity of the AQLQ12+ in adolescents, internal consistency was similar in the two age groups (Table 4) and correlations between each domain of the AQLQ12+ and other measures of asthma clinical status were also very consistent in the two age groups (Tables 5 and 6).

**Table 4:** Standardised version of the Asthma Quality of Life Questionnaire for 12 years and older (AQLQ12+)

AQLQ12+	Mean Score at baseline			Mean change score during treatment (adjusted means)			Internal Consistency at baseline Cronbach's alpha	
			
	≥ 18 yr	12–17 yr	p value	≥ 18 yr	12–17 yr	p value	≥ 18 yr	12–17 yr
	**Study 1**							
Overall	4.95	5.14	0.063	0.58	0.47	0.17	0.96	0.95
Symptoms	4.87	4.96	0.40	0.65	0.57	0.41	0.93	0.92
Activity limitation	5.09	5.41	0.002	0.52	0.39	0.12	0.91	0.86
Emotional function	5.06	5.11	0.68	0.57	0.46	0.26	0.87	0.77
Environmental stimuli	4.64	4.94	0.028	0.54	0.4	0.16	0.82	0.77
	**Study 2**							

Overall	4.69	4.72	0.82	0.66	0.59	0.52	0.97	0.97
Symptoms	4.68	4.64	0.73	0.73	0.67	0.60	0.94	0.95
Activity limitation	4.81	4.96	0.25	0.57	0.51	0.57	0.92	0.91
Emotional function	4.74	4.68	0.70	0.76	0.62	0.27	0.86	0.91
Environmental stimuli	4.24	4.43	0.24	0.57	0.56	0.96	0.84	0.88

**Table 5:** Cross-sectional construct validation (Baseline) (Pearson correlation coefficients)

	**Study 1**					
AQLQ12+	Age	FEV_1_% pred	PEF	Symptoms	Night waking	Rescue bd
Overall	≥ 18 yr	0.15	0.29	-0.50	-0.50	-0.35
	12–17 yr	-0.11	0.13	-0.37	-0.38	-0.21
Symptoms	≥ 18 yr	0.14	0.22	-0.53	-0.54	-0.39
	12–17 yr	-0.11	0.15	-0.34	-0.40	-0.23
Activities	≥ 18 yr	0.14	0.32	-0.47	-0.45	-0.29
	12–17 yr	-0.13	0.15	-0.37	-0.35	-0.16
Emotions	≥ 18 yr	0.15	0.25	-0.36	-0.35	-0.28
	12–17 yr	-0.10	0.05	-0.28	-0.27	-0.23
Environment	≥ 18 yr	0.07	0.24	-0.34	-0.36	-0.22
	12–17 yr	-0.01	0.06	-0.25	-0.25	-0.12

	**Study 2**					
AQLQ12+	Age	FEV_1_% pred	PEF	Symptoms	Night waking	Rescue bd

Overall	≥ 18 yr	0.06	0.23	-0.49	-0.47	-0.35
	12–17 yr	0.03	0.37	-0.46	-0.39	-0.28
Symptoms	≥ 18 yr	0.06	0.17	-0.56	-0.54	-0.42
	12–17 yr	0.04	0.32	-0.49	-0.42	-0.32
Activities	≥ 18 yr	0.06	0.28	-0.40	0.41	-0.28
	12–17 yr	0.05	0.35	-0.42	-0.41	-0.21
Emotions	≥ 18 yr	0.06	0.18	-0.36	-0.36	-0.25
	12–17 yr	-0.02	0.36	-0.32	-0.31	-0.25
Environment	≥ 18 yr	0.03	0.21	-0.34	-0.34	-0.23
	12–17 yr	-0.01	0.40	-0.43	-0.36	-0.25

**Table 6:** Longitudinal construct validity (Baseline – End of study) (Pearson correlation coefficients)

	**Study 1**					
AQLQ12+	Age	FEV_1_% pred	PEF	Asthma symptoms	Night-time awakening	Rescue medication use
Overall	≥ 18 yr	0.3	0.33	-0.43	-0.42	-0.39
	12–17 yr	0.06	0.29	-0.33	-0.2	-0.24
Symptoms	≥ 18 yr	0.32	0.34	-0.46	-0.46	-0.43
	12–17 yr	0.03	0.3	-0.33	-0.26	-0.25
Activity limitation	≥ 18 yr	0.27	0.28	-0.39	-0.37	-0.33
	12–17 yr	0.08	0.26	-0.33	-0.18	-0.21
Emotional function	≥ 18 yr	0.25	0.29	-0.3	-0.31	-0.33
	12–17 yr	-0.06	0.12	-0.22	-0.11	-0.2
Environmental stimuli	≥ 18 yr	0.16	0.19	-0.25	-0.2	-0.2
	12–17 yr	0.19	0.3	-0.21	0	-0.16

	**Study 2**					
AQLQ12+	Age	FEV_1_% pred	PEF	Asthma symptoms	Night-time awakening	Rescue medication use

Overall	≥ 18 yr	0.21	0.41	-0.51	-0.41	-0.37
	12–17 yr	0.23	0.24	-0.45	-0.3	-0.2
Symptoms	≥ 18 yr	0.23	0.4	-0.56	-0.47	-0.43
	12–17 yr	0.26	0.29	-0.47	-0.31	-0.24
Activity limitation	≥ 18 yr	0.17	0.36	-0.43	-0.33	-0.3
	12–17 yr	0.23	0.15	-0.44	-0.28	-0.1
Emotional function	≥ 18 yr	0.14	0.33	-0.4	-0.28	-0.27
	12–17 yr	0.1	0.17	-0.21	-0.22	-0.23
Environmental stimuli	≥ 18 yr	0.17	0.36	-0.32	-0.28	-0.2
	12–17 yr	0.16	0.23	-0.48	-0.22	-0.15

## Discussion

The results of this analysis have shown that measurement properties of the AQLQ12+ in both adolescents and adults are very similar and that the AQLQ12+ can therefore be used in adult clinical trials that include adolescents.

Since only one word change was needed to make the AQLQ(S) to be suitable for adolescents and because both the original AQLQ and the AQLQ(S) have undergone extensive validation in adults [7-11], we have considered the AQLQ12+ in adults (≥18 years) to be the gold standard with which to compare the measurement properties of the AQLQ12+ in adolescents. In both studies at baseline, there was no evidence of any differences in the overall or domain scores except for the activity limitation and environmental stimuli domains in study 1, where small but clinically unimportant differences were observed (the minimal important difference for the AQLQ is 0.5 on the 7-point scale [12]). Changes in scores during treatment and internal consistency were very similar in both age groups in both studies. These data strongly support the validity of the AQLQ(S)12+ in adolescents. There was a very slight tendency for correlations with other clinical indices to be slightly lower in adolescents but this is most likely attributable to a slight difference in the relationship between quality of life and clinical asthma in the two age groups. Even if this is not the reason, the tendency is so small that it not sufficient to suggest lack of validity of the AQLQ12+ in adolescents.

The results of this analysis should not be interpreted to mean that the AQLQ12+ is the most appropriate asthma-specific quality of life questionnaire for adolescents. To evaluate the impact of asthma on individual adolescents in the clinic or to estimate the effect of interventions on adolescents alone, it would be wise to continue to use an instrument that has been specifically developed for this age group. The PAQLQ [2], for instance, includes all the problems that children between 7 – 17 years have identified as important and uses the words that they are most likely to use.

## Conclusion

The results of this analysis suggest that the AQLQ12+ is valid for measuring asthma-specific quality of life in adolescents 12–17 years. The similarity of the measurement properties of the AQLQ12+ in patients 12–17 years and over 18 years provides evidence that data from the two groups can be combined for analysis of adult clinical trials and surveys that included patients 12 years and older.

## List of abbreviations

AQLQ Asthma Quality of Life Questionnaire

AQLQ(S) Standardised version of the Asthma Quality of Life Questionnaire

AQLQ12+ Standardised version of the Asthma Quality of Life Questionnaire for 12 years and older

FEV_1 _Forced Expiratory Volume in 1 second

PAQLQ Paediatric Asthma Quality of Life Questionnaire

## Authors' contributions

EFJ: Design of the analysis, interpretation of data, primary author of manuscript.

KS: Statistical analysis, development of study question, drafting of manuscript.

ACM: Development of study question, drafting of manuscript, study co-ordinator.

ES: Development of study question, drafting of manuscript.

All four authors have played a major part throughout the entire study process from the development of the study question to the revision of the final manuscript. Each of the four authors has made a significant contribution at each phase of the study.

All four authors have reviewed and approved the final version of this manuscript.
